# The prognostic significance of p63 and Ki-67 expression in myoepithelial carcinoma

**DOI:** 10.1186/1758-3284-4-9

**Published:** 2012-03-27

**Authors:** You-Hua Jiang, Bo Cheng, Ming-Hua Ge, Gu Zhang

**Affiliations:** 1 Department of Thoracic Surgery, Zhejiang Cancer Hospital, and The Affiliated Hospital of Zhejiang Chinese Medical University, Hangzhou, Zhejiang 310022, China; 2 Department of Pathology, Clinical Pathological Quality Control Center of Zhejiang Province, Zhejiang Cancer Hospital, and The Affiliated Hospital of Zhejiang Chinese Medical University, Hangzhou, Zhejiang 310022, China; 3 Department of Head and Neck Surgery, Zhejiang Cancer Hospital, and The Affiliated Hospital of Zhejiang Chinese Medical University, 38Guangji Road, Banshan Qiao, Hangzhou, Zhejiang 310022, China

**Keywords:** Myoepithelial carcinoma, Prognosis, p63, Ki-67

## Abstract

**Background:**

Myoepithelial carcinoma is a rare tumour. The clinical and biological behaviours of these tumours are variable. Although many factors have been evaluated as potential prognostic indicators, including clinical stage, site and size of the tumour, high proliferative activity, extensive invasion into the surrounding tissue, perineural permeation, the abnormal presence of nuclear DNA content, and marked cellular pleomorphism, there are no definite histological features that clearly correlate with their behaviour. Thus, conclusions regarding prognostic factors and ideal treatment may emerge as the number of investigated myoepithelial carcinoma cases accumulate.

**Methods:**

Using immunohistochemistry, expression levels of p63 and Ki-67 were determined in 16 myoepithelial carcinoma samples and correlated with clinicopathological characteristics and patient prognosis.

**Results:**

p63 expression was detected in six of the myoepithelial carcinoma tissues (37.5%) and Ki-67 was detected in five (31.3%). In addition, p63 and Ki-67 expression levels were associated with myoepithelial carcinoma recurrence and metastasis. All six patients with p63-positive expression died due to disease or cardiovascular disease (mean survival time = 50.5 months), and p63 expression was statistically significant with respect to survival (*P *= 0.01). Four patients with Ki-67-positive expression died due to disease or cardiovascular disease (mean survival time = 44.0 months); however, there was no statistically significant difference between Ki-67 expression and survival (*P *= 0.24).

**Conclusions:**

Recurrence and metastasis in myoepithelial carcinomas are more frequent in p63-positive and Ki-67-positive EMCs, and poor prognosis is associated with overexpression of p63.

## Background

Myoepithelial carcinoma (MEC) is a rare tumour that was first described by Stromayer et al. (1975) [1]. MEC consists of atypical myoepithelial cells with high mitotic activity and aggressive growth [2]. MEC is an extremely rare tumour of the minor salivary glands, accounting for less than 1% of tumours of this origin [3]. An English-limited PubMed search revealed fewer than 300 cases of MME, with most reports being single case studies [2-7]. The clinical and biological behaviours of these tumours are variable. Although many factors have been evaluated as potential prognostic indicators, including clinical stage, site and size of the tumour, high proliferative activity, extensive invasion into the surrounding tissue, perineural permeation, the abnormal presence of nuclear DNA content, and marked cellular pleomorphism [5], there are no definite histological features that clearly correlate with their behaviour [3]. Thus, conclusions regarding prognostic factors and ideal treatment may emerge as the number of investigated MEC cases accumulate.

p63 is a p53-related DNA-binding protein that helps regulate differentiation and proliferation in epithelial progenitor cells [8]. Recently, p63 was identified as a novel myoepithelial marker that is variably expressed in MECs [7]. The prognostic value of p63 expression in malignant tumours is controversial. Some studies have shown that p63 expression is a good prognostic marker for patients with human urothelial carcinoma [9], but is not an independent prognostic factor for overall survival of esophageal squamous cell carcinoma [10]. However, other reports have found that p63-positive cases had a worse prognosis in patients with oral squamous cell carcinoma [11], adenoid cystic carcinoma of the salivary gland [12], and Merkel cell carcinoma [13]. There are scant data on the association between p63 expression and the prognosis of MEC.

Ki-67 is another marker of cell proliferation [14] and its prognostic significance has been reported in various tumours, including laryngeal carcinoma [15], salivary gland adenoid cystic carcinoma [16], mucoepidermoid carcinoma [17], hepatocellular carcinoma [18], breast carcinoma [19], and lung carcinoma [20]. However, elevated Ki-67 expression in oral and oropharyngeal squamous cell carcinoma did not predict the prognosis of carcinoma [21].

The aims of this study were to determine the expression levels of P63 and Ki-67 in MECs and to establish if the expression of either marker was predictive of survival.

## Methods

### Patient and tissue samples

MEC cases were sourced from the surgical pathological files of patients treated at Zhejiang Cancer Hospital between 1998 and 2010. The archived tissues obtained from the institutional and consultation files were formalin-fixed and paraffin-embedded. One representative paraffin block from each tumour was selected for immunohistochemical study. Approval for the study was obtained through the Zhejiang Cancer Hospital Institutional Review Board.

Follow-up information on the patients' clinical outcome was gathered, including type of treatment, duration of survival following first treatment, tumour recurrence and metastasis, follow-up treatment, time between first treatment and death, and cause of death.

### Immunohistochemistry

Immunohistochemistry analyses were performed to assess the expression of cytokeratin (CK), alpha-smooth muscle actin (α-SMA), vimentin, S-100 protein, calponin, glial fibrillary acidic protein (GFAP), p63, and Ki-67 in the tissue samples. Dilutions and suppliers for all primary antibodies used in the study are detailed in Table 1. Primary antibodies against the respective proteins were added and incubated overnight at 4°C in a humidified chamber. After rinsing with phosphate buffered saline (PBS), slides were incubated with secondary antibody followed by streptavidin-biotin-peroxidase complex, both for 30 min at room temperature with a PBS wash between each step on a Lab Vision Autostainer 720 (Thermo Fisher Scientific, CA, USA) according to the manufacturer's instructions. All tissue slides were counterstained using haematoxylin and eosin (H&E) staining. Appropriate negative and positive controls were used for each antibody throughout the study, with negative controls omitting the primary antibody. Two pathologists, who were blinded to the clinical outcome and other clinical data, independently evaluated the immunohistochemical studies of p63 and Ki-67. The percentage of neoplastic cells with nuclear staining was calculated. A tumour was considered positive when greater than 10% of the neoplastic cells unequivocally expressed p63 or Ki-67 in the nuclei and negative when less than 10% of the malignant cells stained for p63 or Ki-67.

**Table 1:** Antibodies and Dilutions Used in Immunohistochemical Staining

Antibody	Dilution	Company
CK14	1:125	Dako, Carpinteria, CA, USA
α-SMA	1:5000	Sigma BioSciences, St Louis, MO, USA
Des	1:100	Dako, Carpinteria, CA, USA
Vim	1:100	Dako, Carpinteria, CA, USA
S-100	1:400	Dako, Carpinteria, CA, USA
Calponin	1:200	Dako, Carpinteria, CA, USA
GFAP	1:8500	Novocastra, Newcastle, UK
P63	1:200	Dako, Carpinteria, CA, USA
Ki67	1:100	Dako, Carpinteria, CA, USA

### Statistical analysis

The correlation between immunohistochemical data and clinicopathological features was examined using Fisher's exact test, with a *P*-value of < 0.05 considered to be statistically significant. The survival rate was calculated by the Kaplan-Meier method, and statistical differences were assessed by the log-rank test using the SPSS WIN program package 16.0 (SPSS, Inc., Chicago, IL, USA).

## Results and discussion

### Patients and clinical features

**Figure 1:**
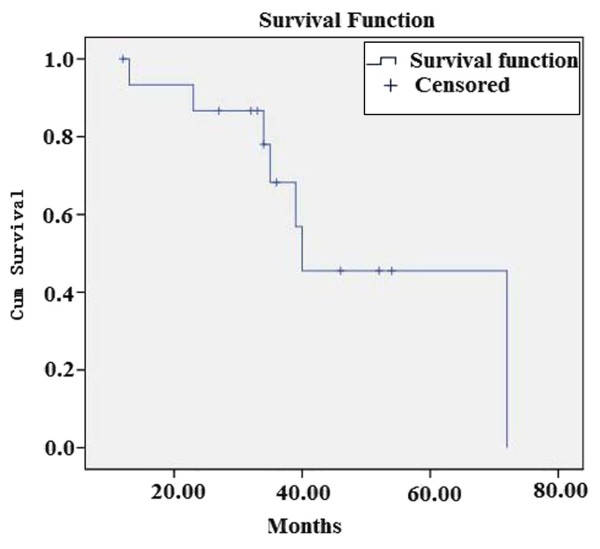
**Using the Kaplan-Meier survival curve, the overall survival rates of 16 patients with MEC at 3 years and 5 years were 68% and 45%, respectively (χ^2 ^= 6.49, *p *= 0.01)**.

Tissue samples from 16 cases of MECs were used in this study. The cases comprised 10 men and 4 women, ages 22-80 years (mean 49.3 years) (Table 2). Tumours arose from the parotid gland (*n *= 4), lung (*n *= 3), maxillary sinus (*n *= 2), nasal cavity (*n *= 2), breast (*n *= 2), submandibular gland (*n *= 1), larynx (*n *= 1), and palate (*n *= 1) (Table 2). Follow-up information was sought for all patients, with the duration of follow-up ranging from 12 to 72 months (mean 36.3 months) (Table 2). Seven patients with complete follow-up had no evidence of recurrence, including two patients with recurrent disease who were treated with additional surgery. Five patients had local recurrences and distant metastases. Sites of metastases included the lung, liver, and brain. Seven patients died of their disease at last follow-up and one patient died due to cardiovascular disease (Table 2). Using the Kaplan-Meier survival curve, the overall survival rates of 16 patients with MEC at 3 years and 5 years were 68% and 45%, respectively (Figure 1).

**Table 2:** p63 and Ki67 Expression, Clinicopathologic Features and Outcome in 14 MECs

Case	Age/sex	Site	Treatment	Recurrence	Metastasis	Follow-up, month (after first time treatment)	P63	Ki67
1	45/M	Nasal septum	Surgery+ postoperative RT	No	No	NED(46 months)	-	-
2	51/M	L Maxillary sinus	Preoperative RT+ Surgery+ postoperative RT+CT	Yes, 17 months	Yes, 20 months, lung	Dead due to MD(23 months)	+	-
3	80/F	L Parotid gland	Surgery	Yes, 12 months	No	Dead due to cardiovascular disease (34 months)	+	+
4	26/F	R Parotid gland	Surgery+ postoperative RT	No	No	NED(27 months)	-	-
5	68/M	R Parotid gland	Surgery+ postoperative RT	Yes, 32 months	Yes, 40 months, lung	Dead due to MD(72 months)	+	+
6	22/M	R submandibular gland	Surgery	No	No	NED(33 months)	-	-
7	62/M	Larynx	Surgery+ postoperative RT	Yes, 38 months	Yes, 32 months, liver	Dead due to MD(39 months)	+	+
8	40/F	L Maxillary sinus	Surgery+ postoperative RT+CT	Yes, 9 months	No	DOD(13 months)	+	¯
9	58/M	L lung	Surgery+ postoperative RT+CT	Yes, 1 months	No	Alive with tumor (32 months)	-	-
10	49/M	Nasal septum	Surgery+ postoperative RT	No	No	NED(12 months)	-	-
11	39/M	L breast	Surgery	No	No	NED(36 months)	-	-
12	65/F	R breast	Surgery	Yes,10 months	Yes, 32 months, lung	DOD (35 months)	+	+
13	44/M	Palate	Surgery+ postoperative RT	Yes,16 months	No	NED(52 months)	-	-
14	49/M	L lung	Surgery+ postoperative RT+CT	Yes,21 months	Yes, 37 months, brain	Dead of due to MD (40 months)	-	+
15	53/M	L lung	Surgery+ postoperative RT+CT	No	No	NED(34 months)	-	-
16	38/F	L Parotid gland	Surgery+ postoperative RT	No	No	NED(54 months)	-	-

*Abbreviations: RT *radiotherapy, *CT *chemotherapy, *L *left, *R *right, *MD *metastatic disease, *DOD *die of disease

### The relationship between immunohistochemical findings and follow-up

**Figure 2:**
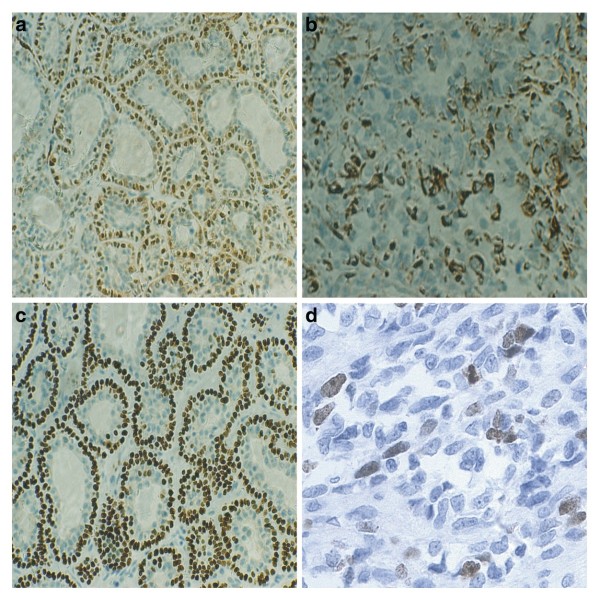
**Immunohistochemical analysis showed that expression of S-100 protein (A), GFAP (B), p63 (C) and Ki67 (D) were positive**.

Of the 16 cases, 15 were immunohistochemically positive for vimentin and calponin staining (93.8%). S-100 protein was immunoreactive in 13 tumours (81.3%) (Figure 2A), immunoreactivity for SMA was seen in 6 cases (37.5%), CK14 reactivity was noted in 12 tumours (75.0%), and GFAP showed positivity in 7 cases (43.8%) (Figure 2B). In addition, limited staining for desmin was observed in 2 cases (12.5%). Using the χ^2 ^test, no statistically significant correlation was demonstrated between the clinical outcomes (recurrence, metastasis, and survival) and these markers.

**Figure 3:**
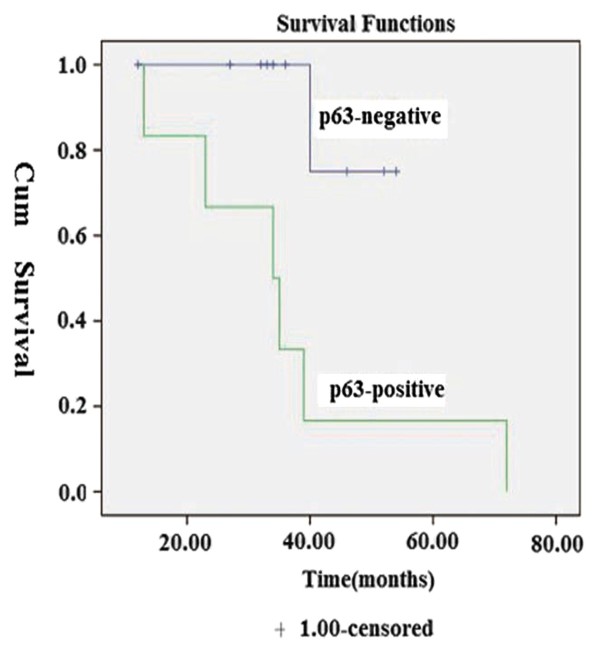
**P63 expression yielded a statistically significant difference with survival (χ^2 ^= 6.49, *p *= 0.01)**.

Immunohistochemical findings for the expression of p63 showed nuclear staining for p63 in 6/16 (37.5%) cases (Figure 2C; Table 2). The mean age of the four p63-positive EMC cases was 61.0 years as compared with 38.2 years for the p63-negative EMCs. All six patients with p63-positive expression had recurrence. A statistically significant correlation was found between p63 expression and the recurrence of EMC (*P *= 0.01). Four patients with p63-positive expression had metastasis and two patients with p63-negative expression had no metastasis. p63 expression showed a statistically significant correlation with metastasis (*P *= 0.03). All six patients with p63-positive expression died due to disease or cardiovascular disease (mean survival time = 50.5 months). P63 expression yielded a statistically significant difference with respect to survival (*P *= 0.01) (χ ^2 ^= 6.49, *p *= 0.01, Figure 3).

**Figure 4:**
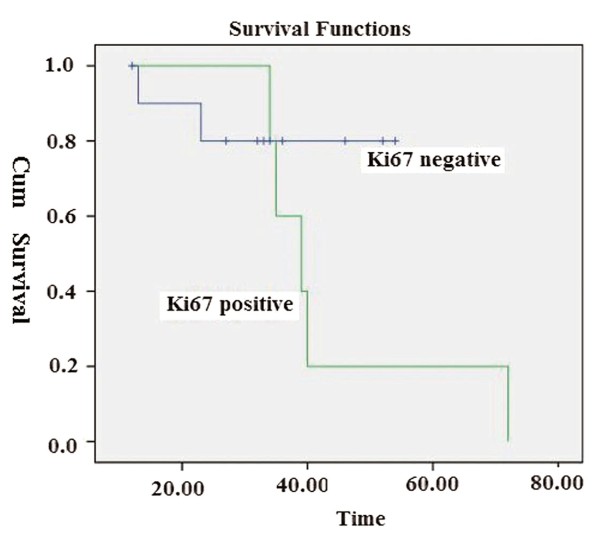
**There was not a statistically significant difference between Ki67 expression and survival (χ^2 ^= 1.38, *P *= 0.24)**.

Nuclear Ki-67 expression was observed in 5/16 (31.3%) cases (Table 2, Figure 2D). The mean age for Ki-67-positive EMCs was 64.8 years as compared with 42.5 years for Ki-67-negative EMCs. A statistically significant correlation was found between Ki-67 expression and recurrence of EMC (*P *= 0.03). Ki-67 expression showed a statistically significant correlation with metastasis (*P *= 0.01). Four patients with Ki-67-positive expression died due to disease or cardiovascular disease (mean survival time = 44.0 months). There was no statistically significant difference between Ki-67 expression and survival (χ^2 ^= 1.38, *P *= 0.24) (Figure 4).

## Discussion

Myoepithelial carcinoma (MEC) is rare, and the causes, clinical behaviour, diagnostic criteria, and outcomes are undetermined [2,5,7,9,10,12]. MEC arises from pre-existing benign lesions, such as pleomorphic adenomas and benign myoepitheliomas^2^, but can also arise *de novo *[1,5].

The precise pathologic definition of MEC remains a matter of controversy because of the morphologic variations in neoplastic myoepithelial cells. Immunohistochemistry is useful for confirming myoepithelial differentiation in MEC, such that all tumours are positive for at least one epithelial marker, cytokeratin or EMA, and most also express either S100 or GFAP [2,7,22]. In the current series, the myoepithelial markers p63 and CK14 were positive in 37.5% and 75.0% of the cases, respectively. The most sensitive myogenic markers were vimentin and calponin (positive in 93.8% of our cases), but these antibodies have little specificity, as they are also expressed in tumours showing smooth muscle or myofibroblastic differentiation [22]. We found nearly all MECs were positive for S-100 protein (81.3%), whereas nearly half were immunoreactive for GFAP (46%), a reactivity profile similar to previously reported result [2]. It has been suggested that assessing cell proliferation activity may be helpful in the differential diagnosis of MEC, and that a Ki-67 labelling index of more than 10% is diagnostic of MEC [2,4]. In the present series, Ki-67 was observed in 5/16 (31.3%) cases. While none of these antibodies are specific for myoepithelial cells, the combination of positive findings support the diagnosis of tumours originating from myoepithelial differentiation [2,4,7,22].

In the present study, the most common site of MECs was the minor salivary gland (7 cases), whereas the major salivary gland was affected in 4 cases. Recurrence and metastasis rates were high (56.3% and 31.3% of cases, respectively). The sites of metastases included the lung, liver, and brain. Seven patients died of their disease at last follow-up and one patient died due to cardiovascular disease. In this study, we analysed the relationship between these markers with clinical outcomes. There were no statistically significant correlations between the clinical outcomes (recurrence, metastasis, and survival) and several markers (vimentin, calponin, S-100, SMA, CK14, GFAP, and desmin).

Although our study included a small number of cases, the results indicated that p63 overexpression is predictive of an unfavourable course. EMCs positive for p63 were diagnosed in older patients (mean age 61.0 years) and p63-negative EMCs were associated with younger patients (mean age 38.2 years). Compared with p63-negative EMCs patients, patients with p63-positive EMCs were showed recurrence, distant metastasis, and poor prognosis (*p *< 0.05). To our knowledge, no reported study has investigated the correlation between p63 expression and outcomes of MECs. Our results were similar to other reports describing different malignant tumours [10-13]. Lo Muzio et al. found that oral squamous cell carcinoma (SCC) patients with p63 overexpression had a poorer survival rate compared to oral SCC patients with a normal pattern of expression (*P *= 0.024). They also suggested that the p63 expression patterns were a reliable indicator of histological grading and an early marker of poor prognosis [11]. Ramer et al. revealed that overexpression of p63 was an independent prognostic factor of adenoid cystic carcinoma of the salivary gland, as determined by multivariate analysis using the Cox proportional hazard model (*p *= 0.012) [12]. Asioli also found p63 overexpression to be a strong independent prognostic factor (*P *< 0.001) in Merkel cell carcinoma, as indicated by multivariate Cox regression analysis [13]. p63 expression has been suggested to have an oncogenic role in the cell proliferation changes observed during carcinogenesis [23]. Conversely, Tuna et al. found that lower p63 expression was correlated with tumour stage, grade, and survival time of urothelial carcinoma (UC) patients (*p *< 0.05) [9]. They suggested that p63 reactivity appeared to be a useful prognostic factor in UC cases [9]. In esophageal squamous cell carcinoma patients, Takahashi et al. revealed that p63-negative expression was associated with poor prognosis and tended to correlate with distant metastasis (*p *= 0.06), and was not an independent prognostic factor for overall survival, as assessed using multivariate analysis (*p *= 0.69) [10]. Therefore, whether p63 expression contributes to prognostic value of these tumours requires further study.

Ki-67 is considered to be a more accurate marker of the proliferative stage of tumour cells than proliferating-cell nuclear antigens (PCNA), and Ki-67 immunoreactivity has been reported to correlate with the prognosis of many cancers [15-20]. In laryngeal carcinoma patients, Ashraf et al. found that tumoural Ki-67 expression correlated significantly with tumour grade (*P *= 0.017) and mitotic count (*P *= 0.001). They suggested that Ki-67 expression in tumoural tissue may be a prognostic marker in patients with laryngeal SCC [15]. Tang et al. revealed Ki-67 expression was an independent prognostic factors of overall survival in salivary adenoid cystic carcinoma, as assesses using multivariate Cox's proportional hazards analysis [16]. In hepatocellular carcinoma patients, Ki-67 was also found to be a significant independent predictor of survival [18]. However, we did not demonstrate the prognostic value of Ki-67 in MECs in the present study. There was no statistically significant difference between Ki-67 expression and survival (*P *= 0.24). However, our results showed that Ki-67 overexpression is related to recurrence and metastasis of MECs. Our study results are in accordance with Li et al. who found that Ki-67 was correlated with lymph node metastasis and was not correlated with prognosis [24]. Our results support the model of carcinogenesis, with increased loss of control of cellular proliferation with the accumulation of genetic alterations in dysplastic lesions.

## Conclusions

To our knowledge, the present study is the first to report the prognostic significance of p63 and Ki-67 in MECs. Despite the small number of cases in the present study, the data suggest that recurrence and metastasis in EMCs are more frequent in p63-positive and Ki-67-positive EMCs, and poor prognosis is associated with overexpression of p63. However, the associations between recurrence, metastasis, and prognosis and the expression of p63 and Ki-67 require further evaluation in a larger series of patients to validate these findings.

## Abbreviations

MEC: Myoepithelial carcinoma; CK: Cytokeratin; a-SMA: alpha-smooth muscle actin; GFAP: Glial fibrillary acidic protein; PBS: Phosphate buffered saline; HE: Haematoxylin and eosin; SCC: Squamous cell carcinoma; UC: Urothelial carcinoma; PCNA: Proliferating-cell nuclear antigens.

## Competing interests

The authors declare that they have no competing interests.

## Authors' contributions

Y-HJ designed the manuscript and performed lung surgery. BC and GZ did immunohistochemistry, and analyzed the results and collected the materials. M-HG performed the head and neck surgery and wrote the manuscript. All authors read and approved the final manuscript.
